# When English clashes with other languages: Insights and cautions from the *Writer’s Craft* series

**DOI:** 10.1007/s40037-021-00689-2

**Published:** 2021-11-03

**Authors:** Lorelei Lingard, Sayra Cristancho, Eva Kathrin Hennel, Christina St-Onge, Marije van Braak

**Affiliations:** 1 Centre for Education Research & Innovation, and Department of Medicine, Schulich School of Medicine & Dentistry and Faculty of Education, Western University, London, Canada; 2 Centre for Education Research & Innovation, and Department of Surgery, Schulich School of Medicine & Dentistry and Faculty of Education, Western University, London, Canada; 3 Institute for Medical Education, Department for Assessment and Evaluation, University of Bern, Bern, Switzerland; 4 Department of Medicine and Centre for Health Sciences Education, Université de Sherbrooke, Sherbrooke, Canada; 5 Department of General Practice, Erasmus Medical Centre, Rotterdam, The Netherlands

In the Writer’s Craft section we offer simple tips to improve your writing in one of three areas: Energy, Clarity and Persuasiveness. Each entry focuses on a key writing feature or strategy, illustrates how it commonly goes wrong, teaches the grammatical underpinnings necessary to understand it and offers suggestions to wield it effectively. We encourage readers to share comments on or suggestions for this section on Twitter, using the hashtag: #how’syourwriting?

At a writing workshop for health professional education (HPE) doctoral students and faculty in the Netherlands, I was explaining how to write more compelling, clear prose. I gave the example of “prepositional pile-up” as a feature writers should avoid. Prepositional pile up, I explained, is too many little phrases that layer on contextual details. I showed an example: “In a study of medical students from rural Australia involved in clerkship rotations at community-based clinics during their final year, we asked …”. The workshop participants laughed. I laughed too, but I wasn’t sure why it was funny. Then someone exclaimed: “Really? But that’s how we write in Dutch! We LOVE our prepositional phrases: the more the better!”

Over the past five years, the Writer’s Craft section has published a series of grammar pearls designed to help writers produce stronger, more memorable research manuscripts [1]. Based in the assumption that writers will maximize their success if they adhere to standard grammatical conventions, these short pearls offer strategies to master sentence structure, strengthen verbs, employ parallel structure, correct punctuation, develop paragraphs, and so on. As the workshop anecdote opening this piece illustrates, however, what’s true for English grammar may not hold for other languages. This may explain why applying the grammar lessons of a Writer’s Craft can be such difficult work. When grammar or style conventions clash across languages, writing well requires both *learning* a strategy and *unlearning* one at the same time. This can create frustration, because the negotiation between clashing conventions is largely tacit, happening beneath the EAL (English as an Additional Language) writer’s surface of consciousness. It is also invisible to the rest of us, meaning that supervisors and co-authors may interpret as *lack* of grammatical knowledge what is actually knowledge of a contradictory grammatical convention in the native language. If writing feedback is based in this interpretation that the writer lacks grammatical knowledge, we’ve got the perfect conditions for imposter syndrome to flourish for the EAL writer. This Writer’s Craft spotlights this challenge with the goal of making explicit and visible some key grammar contradictions. We hope, through this effort, that EAL writers can apply the grammar pearls in the Writer’s Craft series more successfully, and their co-authors and supervisors can target their writing feedback more accurately.

This collaborative piece uses four languages to illustrate this phenomenon: Spanish, French (Canadian), Dutch and German. These languages are a convenience sample that reflects a range of native languages in the HPE scholarly community and allows insight into similarities and differences. Each co-author reviewed all the Writer’s Craft grammar pearls, noting instances where they would need to adapt from the conventions of their native language to accommodate the English convention. The group met to iteratively identify and discuss the most prominent grammar clashes. In what follows, we begin with an at-a-glance summary of the main clashes in all languages, with tips for each to guide writers. Then we focus in on three prominent issues—sentences [2], paragraphs [3], and prepositions [4]—to consider how and why they create challenges for Spanish, French, Dutch and German writers, drawing where possible on published evidence to support our explanations. We illustrate our points using triadic examples that include a native language illustration, a verbatim English translation and the preferred English construction.

Tempted to read only the sections related to your native language? Try not to. HPE is a global field: many of us collaborate and supervise across multiple languages. Greater understanding of one another’s writing challenges can only strengthen these research relationships.

## At-a-glance summary of the main grammatical challenges by language

Based on our analysis, Tab. 1 summarizes the top two grammatical challenges that writers from these languages confront when writing in English. If you are a writer from one of these four languages, this is where your English-speaking colleagues might struggle with your writing. If you are one of their English-speaking co-authors or supervisors, this is where you could help these writers by focusing your feedback more intentionally.

**Table 1:** Key Grammatical Challenges for Spanish, French, Dutch & German researchers writing in English

Language	Grammatical issue	Challenge	Tip
Spanish	Sentence structure	Your tendency may be to write longer sentences and use a variety of synonyms to avoid monotony	Try shorter sentences and word consistency as a strategy to improve clarity
	Prepositions	You may get confused trying to figure out, for instance, when to use “in”, “on”, “at”. In Spanish, you would only use the word “de” for all those three	Spanish has significantly fewer prepositions. You may need to memorize the common English prepositions or use a search engine
French	Sentence structure	Even when you try to write simple and short sentences, it may seem to require more words to do so in French than in English	Avoid long convoluted sentences in English by seeking parsimony: check that all words are essential when critiquing your own writing
	Adjective positioning	You may be used to putting the adjective/qualifier after the noun/subject in French (e.g., blue sky / ciel bleu), so your English writing sometimes does this	Revise each sentence by identifying the noun/subject and adjective/qualifier and verifying that the qualifier precedes the noun as per English word order convention
Dutch	Sentence structure	You may struggle with the position of adjuncts, what a sentence can ‘carry’ in subject position, and the limited freedom in ordering the elements of an English sentence	Avoid ‘heavy’ subject clauses (lots of information in subject position) and make sure the subject position houses the most important information in the sentence. Don’t fling around the parts of the sentence—that can create chaos, rather than cleverness
	Parallel structure	You may tend to use synonyms and variety in sentence structures to ‘polish’ your text. However, variety can compromise clarity and dilute parallelism	Put clarity before variety: avoid synonyms when possible. Try using parallel structure to strengthen your key messages
German	Sentence structure	You may be accustomed to writing longer, more complex sentences that try to build up tension	Aim for short sentences, put the main information first and avoid too many conjunctions
	Paragraphing	Your German paragraphs are supposed to combine several strands of thought, so the principle of paragraph unity can feel foreign	Focus on unity—one idea per paragraph. Start with a topic sentence that clearly signals that idea

## Sentence structure and length

Spanish favors longer sentences than English. The main reason is that Spanish sentences are generally composed of multiple subordinate clauses. Spanish writers use commas, colons or connecting words to join these clauses, rather than separating them with a period. This grammatical feature is called *“extensión del periodo y enlace extraoracional”*—i.e., extension of the period and links between sentences [5]. Here is an example in Spanish with (1) a comma and connector “donde”, (2) a verbatim translation in English that retains the extension thus creating a comma splice and run-on sentence, and (3) a preferred English version.

1. Original Spanish : La investigación cualitativa posee una larga tradición en el area de educación médica**, donde es publicada** en revistas a nivel mundial. (21 words)
2. Qualitative research has come a long way in medical education**, **it is published in journals all over the world. (19 words)
3. Qualitative research has come a long way in medical education. It is published in journals all over the world. (10 words and 9 words)

The preferred version converts the long sentence to two, shorter sentences separated by a period.

In English, simple sentences can strengthen clarity. In Spanish, however, simple sentences tend to be avoided because they are seen as monotonous, repetitive, or lacking rhetorical intentionality [6]. This may explain why Spanish texts tend to be longer than English texts.

The order of the elements of a sentence is another differing feature between Spanish and English. Spanish allows for more flexibility. For instance, the verb can be located before the subject for emphasis. Gender is usually signaled with the past participle verb conjugation, which renders the pronoun unnecessary in some situations. Finally, Spanish adjectives can be placed before or after the noun, but position affects meaning, which is not the case in English.

For French writers, the rules for sentence structure are mostly similar to English rules. French uses the same three types of sentences as English: simple, compound and complex. And the same rules apply for the subject position: it should come early, be in close proximity to the verb, and contain the main idea [7]. The recommended structure in French is “who does what, to whom or what (where, when, how or why)”—*qui fait quoi à qui ou à quoi (où, quand, comment ou pourquoi*) [8]. However, Francophones writing in English may struggle to write crisp sentences because they are accustomed to needing more words to say the same thing in French than in English. Adjective positioning may also pose a challenge, as the French adjectives follow the noun rather than preceding it. In the following examples, word count and adjective positioning are highlighted for the original French (1), the verbatim translation (2), and a preferred English version (3).

1. Dans une *approche de formation* **par compétences**, l’évaluation des apprenants est de plus en plus axée sur les attributs généraux attendus chez les futurs *professionnels* **de la santé** plutôt que centrée spécifiquement sur les connaissances. (35 words)
2. In an *approach to training* **by competence**, the assessment of learners is more and more focused on the attributes generally expected of future *professionals* **of health** rather than centered specifically on their knowledge. (33 words)
3. In a **competency-based** *approach to education*, learner assessment is increasingly focused on the general attributes expected of future **health** *professionals* rather than specifically on knowledge. (25 words)

Dutch writers are encouraged to use short sentences regardless of genre, around 10–15 words [9]. Dutch academic texts have on average much longer sentences, but seem to show a development from long sentences (the German academic tradition) to shorter ones (the English academic tradition). Subclauses (either between commas or between brackets) are very common. The placement of those, as well as the position of adjuncts, is more variable in Dutch compared to English [10].

A striking feature of Dutch sentences that may cause trouble is ‘frontally overloading’ the sentence [10]. Dutch clauses can carry a relatively high word load in subject position, whereas that can overload reader cognition in English. Here is an example of a Dutch sentence (1), translated into English in keeping with its sentence structure (2), and in a more easily readable English counterpart (3).

1. Met heldere academische teksten worden leesbare, objectieve en onderbouwde artikelen bedoeld.
2. By clear academic texts readable, objective and evidence-based articles are meant.
3. By clear academic texts we mean readable, objective and evidence-based articles.

German writers tend to produce longer sentences. German pronouns differ for gender, number and case, which allows writers to refer to subject or objects with clarity even in elaborate sentences. Sentences with up to 25 words are common in scientific texts and are still considered understandable. But the regular use of English as the new lingua franca in science is effecting German scientific texts, with a 20th century trend towards lower complexity and shorter sentences [11, 12].

German writers also tend to frontally overload. Our version of the Dutch example above would be: Mit klaren akademischen Texten sind lesbare, objektive und evidenzbasierte Artikel gemeint. The elements of a sentence are put in rising order of importance, often beginning with an adverbial construction, building up tension, leading to the subject at the end of the sentence [12]. This “inverted word order” is even considered typical for scientific texts. Consider the following example in original German (1), a verbatim translation (2) and a preferred English version which drops the adverbial construction (3):

1. Beim Schreiben eines Satzes sollte darauf geachtet werden, dass die wichtigste Information am Ende steht.
2. When writing a sentence, it should be kept in mind that the main information is at the end.
3. The main information should be at the end of a sentence.

## Paragraph structure

The Writer’s Craft on paragraphing teaches the principles of unity and coherence, where one idea per paragraph is developed by starting with a topic sentence signalling the main idea, developing that idea through body and token sentences, and concluding it with a wrap sentence [3]. This convention translates readily for writers from Spanish and French-language origins. However, Dutch writers may notice some key differences with this approach, and German writers may be baffled given that their approach to paragraphing is quite different.

Dutch paragraphing (not to be confused with the Dutch *paragraaf*, which is a coupling of paragraphs or *alinea’s *into a coherent section) is mostly similar to English conventions. There is one significant difference, though. Dutch paragraphing allows for inverting the conventional structure of the topic sentence followed by elaboration with examples or explanation followed by conclusion [13]. Inverted paragraphs might start with an anecdote illustrating the topic of the paragraph that will be named in the last sentence of the paragraph (see e.g., [14]). The idea is to build tension and enliven the text.

German writers do not learn paragraphing the way it is taught in English [15]. German writers would likely condense the content of a paragraph into fewer sentences. Elaborating around a single idea in a paragraph, therefore, needs to be a conscious effort. Furthermore, putting the main idea into the first sentence of a paragraph feels foreign, because in German writers are more open to encircling the topic and sometimes even finishing with the main idea. These differences reflect the fact that English and German scientific texts come from two different styles of writing: saxon and teutonic [16]. The more linear saxon style presents one thought per paragraph, while the more digressive teutonic style weaves several thematic strands across paragraphs and is more likely to emphasize bridging sentences than topic sentences.

## Prepositions

Spanish writers have fewer prepositions at their disposal compared to the dozens of prepositions used in English to determine the exact location in time and space of an object. For instance, in cases where English distinguishes between “in”, “on,”, “of”, “from” and “at”, in many cases Spanish just uses the words *de *or *en*. Because of this, Spanish writers need to pay careful attention to their use of prepositions when writing in English. English prepositions are not interchangeable: e.g., participants are **in** a study, not **on** a study; a medical school is **at** a university, not **in** a university. Usage, rather than rules, tends to dictate what is correct.

French writers are familiar with the instruction to avoid prepositional pile up in scientific writing. There are many simple and complex prepositions to choose from, arguably more than in English due to contracted prepositions (*prépositions contractées) *which are gender and number specific [7]. The goal is to find the right words, rather than more words. As the example below illustrates, a key challenge for the French writer is using the correct English preposition when they are not as specific as the ones available in French:

1. II a décidé de poursuivre sa carrière médicale **en** milieu académique **à** Paris.
2. He decided to pursue his medical career **in** an academic setting **at** Paris.
3. He decided to pursue his medical career **in** an academic setting **in** Paris.

Probably every young Dutchy has once been corrected in English class for saying that they are “on school” (from the Dutch “Ik zit *op *school”, “I’m at school.”). But in scientific writing, the problem is less the choice of the right preposition and more the extent of use. Unlike English, Dutch writing allows to pile up prepositions to great heights. There is still a point at which a pile up of prepositions drains the power of the message, but the threshold is higher than in English. The following advice is given on a website for writing tips for students of a university and college in Amsterdam: “Houd de vaart erin—vermijd te veel voorzetsels.” [Keep up the tempo—avoid too many prepositions] [17]. That is, don’t produce sentences like:

1. Vaak wordt aangenomen dat het verbeteren van de kwaliteit van het onderwijs dat docenten binnen het medisch onderwijs geven leidt tot een verhoging van het leereffect bij studenten.
2. Often it is assumed that the improvement of the quality of the education that teachers give within medical education results in an increase of the learning effect for students.
3. It is often assumed that improving the quality of teachers’ teaching in medical education results in increased student learning.

German writers may find themselves wondering why the Dutch example needed to be improved. German also has an abundance of prepositions and an openness to piling them up. An almost literal German translation of the Dutch structure (1) at the beginning of this manuscript would sound fine:

In einer Studie mit Medizinstudierenden aus dem ländlichen Australien, die in ihrem Abschlussjahr in Rotationen an Community-Based-Clinics eingesetzt waren, fragten wir ….

Thus, like Dutch writers, German writers may need to watch how many prepositional phrases they use, keeping in mind that the threshold is lower for readers perceiving that their English prose is sluggish.

## Conclusion

For non-native English writers producing scientific manuscripts, success is not simply a matter of learning the English grammar rules and applying them. These writers often *have* learned the English rules (sometimes better than native English speakers!) but they struggle with clashes between these rules and the learned conventions (grammatical and cultural) of their first language [15]. This situation can be exhausting for writers. It also creates fertile ground for imposter syndrome to blossom, particularly when those giving feedback on the writing assume that the problem is a lack of rule-based knowledge. We hope that this brief review of recurring challenges for Spanish, French, Dutch and German writers will normalize the struggle EAL writers experience, will help direct their attention to areas that are most likely to trip them up, and will sensitize their co-authors and supervisors to these grammatical and stylistic complexities.
